# New York Academy of Medicine

**Published:** 1898-08

**Authors:** 


### NEW YORK ACADEMY OF MEDICINE.

### Section in Orthopaedic Surgery, Meeting of March 18, 1898.

*Congenital Dislocation of the Hip.*—Dr. R. Whitman presented a little girl two and a half years of age on whom he had operated for congenital dislocation of the left hip when she was 18 months old. The method followed had been the bloodless operation of Lorenz, and the plaster-of-Paris bandage had been finally removed last November. In the absence of any trace of deformity or disability it was impossible to detect any difference between the two sides, and the cure was evidently perfect. He thought it was the first cure attained by this method in New York.

Dr. T. H. Myers reported that he had seen last week the girl on whom he had operated at the age of three and one-half years in January, 1895, by the method of Paci. The joint was firm with no telescoping. There was no limp, and the child runs, jumps and hops with perfect freedom. He thought it was the first successful application in this city of Paci's method. [See report of Dr. Myers' case and discussion in the TEXAS MEDICAL JOURNAL, June, 1897, pp. 676, 677.—ED.]

Dr. A. M. Phelps said that in the patient exhibited there was a perfect reduction, but it was probably a case of dislocation at birth in a child in whom the acetabulum was normal. He did not believe that the bloodless forcible reduction was a good method. After a child had passed the second year, the head was developed, the actabulum was undeveloped and the capsular ligament was drawn out and constricted like an hour-glass, making reduction mechanically impossible. There was no reduction—simply the conversion of a posterior into an anterior dislocation. The only way was to make an acetabulum and put the head of the bone into it.

Dr. G. R. Elliott said that the acetabulum in these cases was fairly developed in children up to four years of age. The head was felt as it was forced over the border of the socket, it was felt to be retained, and it could be easily dislocated again. If it was retained by fixing the limb at a proper degree of abduction it could not get out of its position, being held by the ligaments and muscular structures, and the probability of its leading to a more perfect acetabular development was greater than after an operation in which the ligaments and muscles had been cut.

Dr. Whitman said that the head of the bone was capable of making an acetabulum and that a rudimentary acetabulum existed in nearly all cases as was proved by the observation that when the head of the femur was pushed in place it stayed there. When the dislocation was anterior, which was not usually the case; the bone should be twisted around.

*Coxa Vara.*—Dr. A. Whitman also presented a boy 11 years of age who had the waddling gait and lordosis of congenital dislocation of the hip. It was, however, a case of double coxa vara with prominent and elevated trochanters. There was free flexion and extension but limited abduction. There was no pain or discomfort. Both femoral necks were depressed beyond a right angle with the shaft, but not bent backward, consequently there was no eversion of the limb, otherwise the signs were typical. The trouble began when the boy was four years old and had its origin in rickets. He has been treated for hip disease at intervals for six years. Dr. Whitman had seen several cases in children, one of whom was but 2½ years of age. The affection is therefore not limited to adolescence.

*Deformity of the Tibia—Osteotomy.*—Dr. F. B. Curtis presented a patient on whom he had operated for the anterior bowing of the tibia. The patient, a girl 12 years of age, had been presented and the case discussed at the meeting of November 19th, 1897. At that time the tibia was three inches longer than that of the sound leg, and the circumference of the leg was 1½ inches more than that of the other. The general health had been poor, probably the result of pain. A skiagram showed thickening with some irregularities in the enlargement and an almost complete disappearance of the epiphyseal line due to pressure. The diagnosis had been undetermined. Sarcoma, syphilitic osteitis, necrosis with a sequestrum, and abscess of the medullary cavity had been suggested and considered. After rest in bed for a month and the administration of iodide of potassium, the tenderness had disappeared and the general health was much improved and it became more evident that the local affection was of syphilitic origin. On January 6th, the fibula was fractured and the tibia straightened and shortened by the removal of a wedge measuring over an inch posteriorly and two inches on its anterior surface. The bone was found to be roughened on the surface and the central canal had disappeared.

The bone was hard, but not so hard as cortical bone in the adult. It was of the same consistency all the way through and was pronounced by a pathologist to be moral in structure. The subcutaneous soft parts were so voluminous that the skin was with dificulty made to cover the wound. Later two long incisions were made on either side of the wound and the skin was dissected up and drawn over the bone. Thiersch grafting was done on February 22nd. The result was a fairly good leg. The bone was of normal length and there was no tenderness. Ability to walk well had not been acquired as the patient had been out of bed only a week.

Dr. T. H. Manly said that the gross appearances were those of malignancy limited to the hard tissues, but with an obvious tendency to infiltrate into and involve the soft parts. The osteoplastic procedure had gained all that could be desired in reducing the length of the limb, but he believed that further trouble was sure to follow and would be interested in the progress of the case.

Dr. Phelps believed that the condition was due to congenital syphilis.

*Multiple Osseous Tuberculosis.*—Dr. V. P. Gibney presented a boy whose previous history was rather obscure. Early in 1897 the left limb had been amputated for "consumption" of the knee. He had been under treatment since last May. There was a focus in the shaft of the left humerus which had been operated on several times and also one on the right elbow. In the latter had been found streptococci, staphylococci and micro-organisms resembling diphtheria bacilli, but no tubercle bacilli. Recently there had been beginning anchylosis of the jaw. A previous diagnosis of multiple sarcoma had been made but it was more than probable that the foci encroaching on the joints were tuberculous.

*Radiograph Showing Osteitic Area.*—Dr. Myers exhibited a radiograph which showed an area of diminished density within the head of the radius and increased density about it. A sclerosing osteitis probably surrounded the site of a caseous focus which had been curetted. After many trials this was the first success he had made in locating a diseased area by the X-ray.

*Pott's Disease Treated by Forcible Reduction of the Deformity.*—Dr. Gibney showed a boy 12 years of age who had had Pott's disease as long as he could remember. There had been no previous treatment. The kyphos had been very marked. On

March 1st, 1898, a moderate degree of force under an anæsthetic had reduced the kyphos a good deal, the parts yielding easily, and a plaster-of-Paris corset was applied in the prone position, from the pelvis to the axillæ. He was kept in bed for three days much against his wishes, and since then has been playing about the yard. There was absolutely no reaction. Another boy, 6 years of age, was presented wearing a plaster of Paris corset after forcible reduction of a well-marked kyphosis. Previous treatment had by apparatus, jackets, etc. The disease in the dorsal region had been long since arrested. The projection had been considerably diminished by an amount of force not greater than in the first case. During the operation his respiration became rather labored and the anæsthesia was discontinued. The only reaction was a slight slowing of the pulse after the operation and on one day since. The patients were presented to show that deformities can be materially reduced by this method without reaction or any immediate bad results. In after treatment it was not necessary to fix the head and shoulders. If the plaster is brought well up there would be no recurrence. The English surgeons were advocates of the steel apparatus; they criticise the French who put their patients up in cotton covered with plaster of Paris. There were plaster jackets and *plaster jackets*. If too much cotton were used a good fit would be impossible, the parts would recede and the jacket would become loose. If the plaster was properly applied it would give no trouble. The fear that forcible correction would induce tubercular action in the meninges or elsewhere was not well founded. In an experience of years in the forcible correction of deformities of the hip it had been the rarest thing in the world to get any dissemination of the bacilli.

Dr. Phelps presented a girl seven years of age wearing a plaster-of-Paris jacket after forcible reduction under ether of an extremely large kyphos. The disease had been of four and one-half years duration, and was between the sixth and ninth dorsal vertebræ. A jacket had been worn for four years. The operation seemed very cruel, and had been undertaken with fear and trembling only partially dissipated by the favorable reports of French operators. The kyphos had been nearly all reduced after so much snapping and cracking that it was thought the child's back was broken. There was no reaction, and the patient was up and about in less than four days. The procedure was applicable to the early stages of the disease. In the presence of a large kyphos or anchylosis or abscess it was a dangerous method.

Dr. R. W. Townsend related a case in which an inconsiderate resort to this operation would have been disastrous. A girl three and one-half years of age was under treatment upon an open frame for disease in the upper dorsal region. There was a cough and impeded respiration, and other symptoms of bronchitis, followed rather suddenly by asphyxia and death. Autopsy showed a retro-pharyngeal abscess in the median line directly over the vertebral column and extending to the right. There was no pressure on the trachea, which was normal in size and not flattened. Numerous enlarged glands had pressed on the recurrent laryngeal nerve, and caused paralysis of the vocal cords. The second dorsal vertebra was so much diseased that the finger was pushed right through to the spinous process. Forcible reduction would have ruptured the abscess or done some damage to the bone. The dangers of the operation were readily realized. The proceedure might give good results in suitable cases, but it should be well tried before being widely recommended.

Dr. Myers had not as yet heard of any cure as the result of this procedure. The cases should be very carefully selected, and care taken to ascertain that no abscess was present. The operation was dangerous, and results should be waited for before the method should be commended at all. The protection given to the spine after the operation should be most perfect.

Dr. H. L. Taylor could not think well of this method without the light of further experience. The tendency had been to make the procedure much less radical than it had been at first, when reduction of the deformity sometimes called into action all the strength of the operator with perhaps resection of the projecting spinous processes, and in suitable cases excision of wedges of bone. In some instances the spinous processes were wired together after reduction, and it was considered important to encase the head of the pelvis in the plaster-of-Paris jacket. With this evident tendency towards simplification of the treatment, it remained to be seen how much of the original operation would remain after the method had been well tried. It was safe, thus far, in the hands of experts, but it would be dangerous to encourage the general practice of the method.

Dr. R. S. Sayre said that if the diagnosis were made before the kyphos appeared there would be no necessity for this operation. He thought that if it could be determined in advance which cases could be straightened without damage, this operation could be readily accepted. In some cases there were no vertebral bodies left and the column was held together by the spinous and transverse processes. In other cases the bone was so diseased that forcibly straightening the spine would produce gaps between the vertebræ leading to the production of abscesses. It was extremely doubtful whether this method should be employed. In any event the cases should be most carefully selected and there should be no elevation of temperature and no morbid action present.

Dr. Manley thought that the forcible correction of this deformity in appropriate cases was justifiable, to be followed by some form of thoracic support after correction.

Dr. Elliot said that the two cases of forcible correction followed by death had been recently reported in the *British Medical Journal.*

Dr. A. B. Judson had seen no reason for not being satisfied with treatment by the use of the steel brace. Patients with Potts' disease suffered so much inevitable daily traumatism in standing and walking that the injury accompanying the method under discussion would not seem to be necessarily fatal or even dangerous. The question was whether it was wise to add to the unavoidable and habitual traumatism. If we could restore the curves and strength and mobility of the spine, almost any treatment would be accepted. But it could not be hoped to carry recovery to that acceptable point. Moreover, it was very doubtful whether the consolidation would come to our aid at the opportune moment to secure the improvement in shape made by the forcible reduction.

Dr. Phelps had looked up the literature of the subject. On the one hand it had been stated and demonstrated by radiographs that bone had been reproduced in cases in which there was wide separation after reduction, and one operator had reported 204 cases with no deaths and no accidents. On the other hand, other operators had reported many relapses, sometimes with paralysis, a number of deaths had been reported, the kyphos had been reduced in a cadaver with rupture of an abscess and in another subject with a fracture of the vertebra. Some investigators are enthusiastic in favor and others condemn in round terms. Although there was probably a field for operation it was necessary to proceed slowly.

Dr. Gibney said Dr. Townsend's patient was an exceptional one. Most patients seen offered no contra-indication to the operation. He had not found that patients with a deformity of the spine were cheerful at the prospect of going through life with it. They were morose, and felt that Nature had treated them harshly and it was necessary to do something for them. If he had a child with such a deformity he would welcome almost anything which promised relief. He understood the dangers of the operation and was opposed to its wholesale performance. While fully appreciating the importance of what had been said he thought that clinical facts were also entitled to weight. He believed that deformities could be materially reduced by the method but that it should be done gradually at several sittings rather than all at once.

*Lateral Curvature Treated by Forcible Reduction.*—Dr. Gibney also presented a patient, a girl 14 years of age, affected with lateral curvature of the spine whose parents had urged that something be done to correct the deformity, being willing even to have a section done. Under an anæsthetic a twisting motion was employed for some five or ten minutes and the patient was then put into plaster-of-Paris. She was treated three times. There had been no reaction. She had gained one and three-fourths of an inch in height and the back was in a much better position than it had been before.

*An Unusual Case of Potts' Disease.*—Dr. Townsend presented a patient in whom the deformity had not been attended by symptoms. The patient was a girl 11 years of age. Two weeks ago the mother, giving the child a bath, had for the first time noticed a projection which on examination was found to be at the tenth and eleventh dorsal vertebræ and so sharp that the case did not seem to be a favorable one for forcible reduction. The child was in good health and had had no pain or any other symptom and no history of illness. She was extremely active and could stand any amount of jarring. Excepting near the kyphos the spine was very flexible. There was also at the site of the projection a slight deviation to the right but no sign of rotation.

Dr. Gibney recalled the case of a patient who, without pain and with no history of symptoms, presented a similar deformity of apparently rachitic origin. He had intended to apply forcible reduction.

*Cervical Abscesses Complicating Potts' Disease.*—Dr. Myers presented a boy seven years of age with Potts' disease of the third and fourth dorsal vertebræ complicated with an abscess discharging in the neck behind the right sterno-cleido-mastoid muscle. The treatment had illustrated the effect of the supine position in securing good drainage. The spine had been protected by a brace, but the discharge was profuse and the temperature varied between 100 degrees and 103 degrees, when he was placed supine in bed without a pillow. In two weeks the temperature dropped 2 degrees. He was then allowed to leave his bed, and the temperature soon rose to the former level. He was then returned to bed and the temperature subsided as before. The discharge gradually decreased, and when it had nearly ceased he was allowed to be up. His general health was entirely restored and there had been no discharge for several months.

Dr. Townsend presented a girl three years of age with Potts' disease of the upper cervical vertebræ. There was an abscess the size of a hen's egg extending around the outer side of the sterno-cleido-mastoid, about half being deep seated, the remainder superficial. Two-thirds of the abscess was posterior to the muscle, and over the swelling were a number of enlarged veins. The patient had been put on a frame and the abscess would be opened without undue delay by an incision back of the muscle.

Dr. Phelps urged the importance in similar cases of early operation to prevent rupture into the pharynx and the occurrence of tuberculosis from the flowing of the infective material into the larynx during sleep.

*Malignant Disease of the Spine.*—Dr. Taylor presented a man 47 years of age, in poor general condition, and giving a history of very severe pain in the lower part of the back for eight months. In that time he had lost much flesh and had been disabled by limitation of motion in the lumbar region of the spine and difficulty in locomotion and other movements of the body. The upper part of the back was rounded and the spine of the first lumbar vertebra was prominent and deviated to the left. There was a well defined area of sweating in the right loin probably from invasion of a sympathetic ganglion and vaso-motor paresis. This symptom was not present in tubercular disease of the spine but he had observed it before in cases of malignant spinal disease. Partial relief would probably follow the application of a jacket, and morphine would be given to control the pain.

Dr. Sayre suggested the use of the actual cautery to temporarily relieve the pain.

*Apparatus for Forcible Extension.*—Dr. Elliott exhibited an instrument which he had devised for forcible extension, especially for the reduction of the congenitally dislocated hip. It was light and inexpensive and consisted of a small rectangular frame about 6 inches wide and 15 inches long. In the centre of the long axis was an extension screw. To a cross-bar on this extension screw was attached preferably the ends of a skein of yarn which had previously been adjusted to the ankle of the patient. The frame could be affixed to any table or bed and the force employed, the patient being firmly held by an assistant, could be regulated at will and, if desired, measured in pounds. The instrument, besides being used for reduction of dislocation of the hip as described, could also be conveniently used in the forcible reduction of the spinal column in cases of angular curvature, the proper adjustments being easily made.

MEETING OF APRIL 22, 1898.

*The Treatment of Rachitic Deformities.*—Dr. R. H. Sayre read a paper with this title, of which the following is an abstract.

Patients affected with well-marked rachitic deformities would not "out-grow" them. Treatment should be active, and should vary with the pathological stages of the affection. In acute rickets cod-liver oil would be of great benefit and phosphorus in the elixir of the National Formulary would yield good results, given three times a day in doses as large as the fiftieth of a grain. When the bones were soft they should be subjected to manipulation, and the child should be kept in the recumbent position, which is best done, if there are spinal deviations which require special attention, by the use of the wire cuirass. The two great aids of fresh air and sunshine would thus be invoked and would be not less effective than treatment by the use of drugs. Instruments should be applied, in order to retain the improved position secured by manipulation. In decided knock-knee or bow-legs, if the bones are not too hard, the deformity might be corrected by the plaster-of-Paris bandage changed in shape and in the degree of its pressure from time to time by cutting out a section of the plaster at the point of greatest deviation, inserting a wedge in the cut and applying additional plaster to retain what is gained. In similar cases of coxa vara depending on a rachitic process in adolescence, the deformity could be overcome by traction in the recumbent position. When eburnation was established, efforts to correct deformity by manipulation or by instruments would be a waste of time, and the osteoclast or the chisel should be used, the latter when the division was to be made near a joint, and the former when the force was to be applied at a point an inch or more removed from a joint. In some extremely rachitic subjects non-union followed an operation, from delayed formation of new bone cells due to eburnation and impaired nutrition. A not uncommon manifestation of rickets was seen in pigeon-toes, the result of an instinctive turning in of the toes to avoid receiving the weight of the body directly on the weakened tissues of the foot. Injudicious efforts to induce the foot to turn out might lead to flat-foot, and increase the usually present tendency to knock-knee.

Dr. S. Ketch had used phosphorus in the treatment of rickets for twenty years, with excellent results. He preferred a compound syrup of the hypophosphites containing lime, iron, potash and soda, without strychnine. Manual force was of benefit in nearly all cases, especially when the patients were off their feet. When they began to walk, gratifying results would follow the employment of the usual bow-leg and knock-knee apparatus. Unless there were exceptional reasons for protecting the spine, he would not use any restraining apparatus, like the cuirass, for recumbent patients. Time should not be wasted in waiting for the patient to "grow out" of the deformity.

Dr. A. M. Phelps said that at the beginning of the treatment the patient should be taken off his feet, and if the bones were not hard, we should bend the bones, producing, perhaps, a green-stick fracture, and thus straighten them by manual force. Bones that had become sclerotic required osteoclasis or osteotomy. The latter should never be done in children under twelve years of age, unless demanded by some unusual condition, and in all cases osteoclasis should, as a rule, be preferred to osteotomy. Non-union was found only after osteotomy, and it was attended with some danger, deaths have been reported, whereas these accidents never occurred from osteoclasis.

Dr. Ketch opposed operative procedure in the early stage. The experience of the past and of the present day showed that manual force and mechanical treatment were sufficient to effect a cure. A speedy rectification of the deformity by an operation was misleading, because the time required by the mechanical treatment after the operation was as long as it would have been if no operation had been done. In no way, except mechanically, could the child be protected against a return of the deformity.

Dr. A. B. Judson said that in orthopædic practice operations should as a rule be avoided. The patients were children and the question of time was unimportant. If pressure were applied in the direction opposite to the deformity and due time and attention were given, the natural growth was a curative agent. If treatment were begun early and the patients were taken off their feet the deformities of rickets especially were curable by mechanical methods alone.

Dr. R. Whitman said that the slight deformities of children should receive more attention than was customary. He had observed that pigeon-toes were as a rule symptomatic of rachitic knockknees or flat-foot and represented an effort of Nature to restrain deformity. Many cases of coxa vara had their origin in infantile rickets. The deformity of the femoral neck was latent in childhood. During adolescence the neck, being from its depression subjected to a greater strain, gave way and deformity and disability followed. In the same way adolescent knock-knee was in many instances an exaggeration of a slight deformity in infancy. In extreme cases of coxa vara he thought that restoration of abduction by division of the neck of the femur was generally required and that in any kind of a case simple extension would not often be effective.

Dr. C. N. Dowd said that after osteotomy above the femoral condyles he had found that the corrected position could be perfectly maintained by carrying the plaster-of-Paris to the upper part of the thigh instead of enclosing the thorax. A towel was put between the knees and the feet were tied together. Cleanliness was thus promoted and the children sat up in bed, ate in comfort from a tray and could play with toys without interfering with treatment during the period when the bone was uniting.

Dr. Whitman preferred the double spica of plaster-of-Paris which insured absolute fixation and in the routine of hospital treatment had not entailed undue inconvenience or unpleasant consequences.

Dr. Sayre said that an early diagnosis followed at once by active treatment when the tissues were soft and ready to yield to moderate pressure would prevent a great deal of trouble at a later stage when eburnation would require that the deformity be reduced by forcible procedures. He thought that a child could not be kept in bed very well without some apparatus. If the cuirass was objectionable, put on a pinafore and fasten it here and there to the bed and thus bring the child to anchor. In serious cases the cuirass or Bradford's frame were a great convenience. Children in them are like little wooden images and they live in them for two or three years, are happy and comfortable and have their toilet made without any great bother to the nurses. Apparatus very useful to hold the bones in position after they had been brought to a straight position but to bring them to a straight position, apparatus was not nearly so efficient as putting them up in plaster-of-Paris. It was simply a question of leverage and he had found that leverage was more acurately applied by the use of plaster-of-Paris than by any other means.

*A Specimen of Congenitally dislocated Hip.*—Dr. G. R. Elliott exhibited a specimen which was removed from a child seven years of age. It showed congenital dislocation of the right hip. During life there was one inch of shortening of the right lower extermity with eversion. Post mortem findings: Gluteal group of muscles somewhat shortened. Pyriformis muscle one-half inch shorter than its fellow and reduced to a tendinous band. Abductor group of muscles somewhat atrophied. The neck of the femur of the affected side was very short, the upper part of the bone consisting chiefly of head and great trochanter. Displacement upward and forward. Capsule thickened, shortened and intact. Head of bone fairly normal and synovial surface in good condition. Ligamentum teres lengthened but size apparently normal. Acetabulum to be examined and reported upon later.

